# Varying Doses of Rare-Earth-Metal-Based Neodymium Zirconate Zinc Sulfide Nanocomposite Disrupt Blood and Serum Parameters, as well as Markers of Oxidative Stress in the Selected Organs of Albino Mice

**DOI:** 10.3390/genes13122262

**Published:** 2022-12-01

**Authors:** Tafheem Khosa, Mourad Ben Said, Zia Ur Rehman, Muhammad Ali, Sania Jamil, Qandeel Fatima, Hafsa Hussain, Rehana Iqbal, Adil Khan, Furhan Iqbal

**Affiliations:** 1 Institute of Zoology, Bahauddin Zakariya University, Multan 60800, Pakistan; 2 Higher Institute of Biotechnology of Sidi Thabet, University of Manouba, Manouba 2011, Tunisia; 3 Laboratory of Microbiology, National School of Veterinary Medicine of Sidi Thabet, University of Manouba, Manouba 2011, Tunisia; 4 Department of Physiology, The Islamia University of Bahawalpur, Bahawalpur 63100, Pakistan; 5 College of Veterinary Sciences, Bahauddin Zakariya University, Bahadur Sub Campus Layyah, Layyah 31200, Pakistan; 6 Department of Botany and Zoology, Bacha Khan University, Charsadda 24420, Pakistan

**Keywords:** Neodymium zirconate zinc sulfide, hematobiochemical analysis, oxidative stress markers, albino mice

## Abstract

Despite extensive industrial use, the biocompatibility of nanocomposites has not been extensively explored. The present study was designed to report the effect of variable doses of a newly synthesized nanocomposite, Neodymium Zirconate Zinc Sulfide, on selective serum and complete blood count parameters and on the oxidative stress markers from the vital organs of albino mice. Albino mice (C57BL/6 strain, 5 weeks old) of both sexes were orally treated for 11 days, either with 10 mg (low dose) or 20 mg/mL saline/kg body weight (high dose) of Neodymium Zirconate Zinc Sulfide nanocomposite. A control group that was not treated with the nanocomposite but with saline solution was also maintained. Data analysis revealed that high-dose nanocomposite-treated male mice had significantly reduced hemoglobin concentration as compared to the control males. Female mice treated with both doses of nanocomposite had higher serum triglyceride levels than controls. High-dose-treated female mice had elevated serum cholesterol concentration compared to their saline-treated controls. Oxidative stress marker analysis from selected organs indicated that concentrations of malonaldehyde (MDA) in the kidney and liver, Superoxide dismutase (SOD) levels in the brain and catalase in the kidney of male mice treated with the nanocomposite were significantly higher than in the control group, whereas SOD in the heart, MDA in the heart and kidney and catalase levels in the kidney were significantly disrupted in female mice compared to their respective controls.

## 1. Introduction

The IIIB group of the periodic table contains seventeen elements that are known as rare Earth elements (REEs) and they are a topic of active research in the current era, as REEs are part of a large number of clinical and industrial applications [1,2]. On the other hand, this extensive REE use has led to extraordinary deposition of these elements in human and animal bodies as well as in the environment through the food chains and webs [3]. Accumulation of REEs beyond safe limits in animals is known as toxicity in the living systems, disturbing their normal neuronal and reproductive physiology [4]. 

Neodymium is a REE material that is widely used in industry nowadays and it is considered to be the fourth largest REE in use, with an average usage of around 7300 tons per year [5]. Neodymium is commonly used in the manufacture of audio systems, magnets, hybrid engines, hard drives of laptops and wind turbines; all of these materials are part of our daily lives [6], while Zinc Sulfide is commonly used in optical sensors, photoconductors and in dielectric filters [7]. Despite these nanomaterials having a variety of uses and advantages, their safe limits and toxicity have never been explored and it is the need of the hour to determine the biocompatibility of these nanomaterials before their large-scale commercial use to counter their adverse effects to organisms and their environment [8,9]. Recently, we reported that the oral supplementation of Neodymium Zirconate Zinc Sulfide results in disturbed exploratory behavior and muscle activity of albino mice [7]. This led us to investigate whether applied doses of this nanocomposite affect the blood cell count, selective serum parameters and oxidative stress markers from the vital organs of albino mice in a sex-specific manner. 

## 2. Materials and Methods

### 2.1. Synthesis of Nanocomposite

Neodymium Zirconate Zinc Sulfide nanocomposite used in the present investigation was previously synthesized in our laboratory via the micro-emulsion method as reported earlier [7].

### 2.2. Experimental Animals 

Albino mice (C57BL/6 strain, 5 weeks old) were used as experimental animals and breeding colony was maintained as described previously [7]. Animals were individually kept in locally manufactured rodent cages during the experiments. 

### 2.3. Experimental Design

Five-week-old mice were divided into three groups. Each group contained equal number of males and females. Group 1 (*n* = 16) orally received the low dose (10mg/mL saline/kg body weight) and group 2 (*n* = 16) orally received the high dose (20 mg/mL saline/kg body weight) of Neodymium Zirconate Zinc Sulfide nanocomposite. The doses were applied once per day for eleven consecutive days. Group 3 (*n* = 16) orally received saline solution for the same duration: the control group. 

### 2.4. Determination of Hematological Parameters

Following the specific dose supplementation, the mice were sacralised under Isoflurane anaesthesia and the blood from each animal was either drawn from the retro-orbital sinus or through the cardiac puncture. A part of blood was preserved in a tube containing EDTA as anticoagulant for the determination of complete blood count parameters by using a complete blood count analyzer (Sysmex, Kobe, Hyogo, Japan) following Akram et al. [10].

### 2.5. Determination of Serological Parameters

A part of blood was collected from each mouse in an Eppendorf tube (without EDTA) and centrifuged at 14,000 RPM for 15 minutes to separate the serum that was later used for the determination of triglycerides, cholesterol, creatinine, albumin, urea and total protein by using a fully automated biochemistry analyzer (Selectra ProM, Paris, France).

### 2.6. Determination of Biomarkers of Oxidative Stress in Organs

Following the sacrifice, brain, liver, heart, lungs and kidney were surgically removed from each mouse, rinsed in isotonic saline solution and stored at −20 °C until each organ was analyzed for the selective markers of oxidative stress.

Superoxide dismutase (SOD) concentrations were calculated according to Noureen et al. [11]. Briefly, a 10% (*w*/*v*) tissue homogenate was prepared in phosphate buffer (0.05 M, pH 7.8) and centrifuged for 15 minutes at 10,000 RPM. Resultant supernatant (0.5 mL) was mixed with 1 mL of sodium carbonate (50 mM), 0.2 mL of EDTA (0.1 mM) and 0.4 mL of NBT (24 µM) in a test tube and mixed by vortexing. Then, 0.4 mL of Hydroxylamine hydrochloride (1 mM) was added to continue the reaction. A reaction mixture without tissue homogenate was run as negative control. Absorbance of SOD was recorded at 560 nm.

Protocol of Saleem et al. [12] was followed for the estimation of lipid peroxidation in mouse organs. Tissue homogenates were prepared in 0.1 M phosphate buffer of pH (7.0). A reaction mixture was prepared by mixing 0.375% thiobarbituric acid (TBA) and 15% trichloroacetic acid (TCA). Then, 300 µL of tissue homogenate and 2 mL of TCA-TBA mixture were mixed in a test tube and a water bath was used to boil it for 15 minutes followed by cooling in ice-chilled water. Tubes were centrifuged at 13,000 RPM for 10 minutes. A supernatant with pink color was produced, the absorbance of which was recorded at 532 nm. 

Protocol of Khadija et al. [13] was applied for catalase estimation. Tissue homogenate (10%) was prepared in 0.01 M phosphate buffer pH (7.0) and centrifuged at 10,000 RPM for 10 minutes. Thus, 0.1 mL of supernatant was mixed with 1.4 mL of reaction mixture that contained 0.4 mL of 2M H_2_O_2_ and 1 mL of 0.1M phosphate buffer. The reaction was terminated after one minute by adding 2 mL of dichromate-acetic acid. A boiling water bath was used to heat the mixture for 10 min that resulted in stable green-color solution due to the formation of chromic acetate. Negative control samples having distilled water in place of supernatant were used. The % inhibition was calculated at 570 nm. 

### 2.7. Statistical Analysis

Statistical package Minitab (version 16, State College, PA, USA) was used for data analysis. All data were expressed as mean ± standard error of mean. Significance level was set at *p* < 0.05. One-way ANOVA followed by two-sample Student’s *t*-test were applied to compare all the parameters of complete blood count, serum and markers of oxidative stress between low- and high-dose Neodymium Zirconate Zinc Sulfide nanocomposite- and saline-treated mice of both sexes.

## 3. Results

### 3.1. Characterization of the Neodymium Zirconate Zinc Sulfide Nanocomposite

X-ray diffraction (XRD) analysis was carried out to confirm the crystal structure and purity of the Pyrochlore and Zinc Sulfide. A scanning electron micrograph (SEM) was used to calculate the particle size of the nanocomposite and the particles were in a range of 14.7–42 nm. The detailed synthesis and characterization of the nanocomposite used in this study are provided elsewhere [7]. 

### 3.2. Complete Blood Count Analysis

One-way ANOVA test results indicated that haemoglobin concentration was the only parameter among complete blood count analysis that varied significantly (*p* = 0.01), when compared between nanocomposite-treated and untreated male albino mice. Male albino mice treated with a high dose of nanocomposite exhibited the least haemoglobin concentrations among all three treatments. One-way ANOVA test results did not reach statistical significance (*p* > 0.05) for any studied parameters when analyzed for female mice). 

Two-sample Student’s t test result indicated that haemoglobin level was the only parameter that was significantly reduced (*p* = 0.02) in low-dose-treated male mice when compared to the control group. All other parameters were varying non-significantly (*p* ˃ 0.05) when they were compared between low- or high-dose nanocomposite treatments and nanocomposite-untreated mice of both sexes (Table 1).

### 3.3. Analysis of Serum Biochemical Parameters

One-way ANOVA results revealed that cholesterol (*p* = 0.01) and triglyceride (*p* = 0.04) concentration were significantly different when compared between nanocomposite-treated and untreated female albino mice. Female mice treated with a high dose of nanocomposite showed the highest concentration of cholesterol and triglyceride parameters. ANOVA test results were not significant (*p* > 0.05) when calculated for male mice.

Results of two sample t test indicated that female mice treated with a low (*p* = 0.03) as well as high dose of nanocomposite (*p* = 0.02) had more elevated serum triglyceride levels than the saline-treated group, while female mice treated with a high nanocomposite dose had higher serum cholesterol concentration (*p* = 0.05) compared to the control group (Table 2).

### 3.4. Analysis of Biomarkers of Oxidative Stress in Vital Organs

One-way ANOVA results revealed that brain superoxide dismutase (SOD) concentrations (*p* = 0.05) and kidney catalase concentration (*p* = 0.03) varied significantly when compared between nanocomposite-treated and untreated male albino mice. Highest SOD and catalase concentrations were observed in high-dose nanocomposite-treated male mice.

Two sample Student’s *t*-test results indicated that malonaldehyde (MDA) concentrations were significantly elevated in the liver (*p* = 0.05) and kidney (*p* = 0.03) of low-dose nanocomposite-treated male albino mice compared to nanocomposite-untreated control mice (Table 3). SOD concentrations were found significantly elevated in the brain (*p* = 0.05) and catalase in the kidney (*p* = 0.03) of male albino mice treated with a high dose of nanocomposite when compared to their control groups. All other studied biomarkers of oxidative stress in vital organs of male mice varied non-significantly (*p* > 0.05) when compared between nanocomposite-treated and untreated animals (Table 3 and Table 4).

One-way ANOVA results showed that catalase concentrations in the kidney (*p* = 0.01) and heart (*p* = 0.03) of female albino mice varied significantly when compared between control, low and high dose of nanocomposite-treated groups.

Analysis of two-sample Student’s *t*-test revealed that SOD concentration in the heart was the only parameter that was significantly elevated in low-dose nanocomposite-treated female mice as compared to their respective control groups (Table 3). For high-dose-treated females, *t*-test results revealed that MDA concentrations in the heart (*p* = 0.03) and kidney (*p* = 0.03) and catalase concentration in the kidney (*p* = 0.03) were significantly decreased as compared to their respective control groups. All other parameters varied non-significantly (*p* > 0.05) in all organs of nanocomposite-treated and untreated female mice (Table 3 and Table 4).

## 4. Discussion

Exposure to rare Earth elements (REEs) is known to induce oxidative stress in living systems, in a manner similar to that induced by other transition elements. REE exposure may lead to growth inhibition, genetic defects and toxicity in vital organs [14,15]. Nanomaterials have the ability to gain entry into living systems through a number of routes: through food, water and inhalation or through dermal penetration [11]. Oral administration of nanomaterials results in their interaction with blood components and they are able to induce cell membrane disturbances. Nanomaterials interact with plasma and their associated proteins, which can lead to various pathophysiological processes [16].

Heavy metals are known to disturb the composition and physiology of the hematological system and environmental exposure to high concentrations of these metals can cause anemia in animals [17]. It was recently reported in a human-based study that with the increasing use of REEs, including Neodymium, their concentration is increasing in e-wastes and reaching the population. Henríquez-Hernández et al. [14] reported higher REE levels in sub-Saharan immigrants and found an inverse correlation between most of these elements and the hemoglobin concentrations of these subjects. In another study, conducted on rabbits, Adu et al. [18] observed a significant decrease in mean corpuscular hemoglobin concentration and mean corpuscular hemoglobin following exposure to variable doses of Cerium oxide in the diet. The studied parameters had an inverse correlation with the applied dose of RRE. During the current investigation, we observed a similar significant decrease in hemoglobin concentrations in male mice after exposure to a low dose of Neodymium containing the nanocomposite (Table 1).

We observed that nanocomposite-treated albino mice exhibited a significant increase in serum triglycerides and cholesterol level (Table 2). There are different trends reported in the literature regarding the effects of REEs on serum biochemical parameters. Similar to our findings, Cheng et al. [19] reported a significant elevation in serum triglycerides in mice that were exposed to lanthanides but, in contrast, Wang et al. [20] documented that chronic oral exposure to variable doses of lanthanum chloride (LaCl_3_) did not affect the serum triglyceride levels of Wistar rats. Similarly, Shin et al. [21] observed no difference in serum triglyceride levels in male rats that were exposed to variable doses of nano-sized La_2_O_3_ for 28 days, as compared to the control group.

Huanga et al. [22] reported that entry of rare Earth ions in the liver results in their accumulation in the liver and, eventually, these ions are transformed into meta-stable hydrogen oxide particles, inducing toxicity.

Nanomaterials are known to induce oxidative stress in living systems by generating reactive oxygen species (ROS), affecting normal gene expression, resulting in a number of disease conditions [23]. In order to counter this ROS-mediated toxicity, cells have an antioxidant system consisting of enzymes and non-enzyme biomarkers of oxidative stress that try to maintain normal cellular functioning [8,24]. In the present study, a significant increase in brain SOD levels was observed in high-dose nanocomposite-treated male albino mice (Table 4). Our results are in agreement with Zhao et al. [25] while exposing mice to lanthanides and reported that Ce^3+^ and Nd^3+^ caused oxidative stress and toxicity in the mouse brain. They documented that lanthanides trigger a cascade of reactions, including lipid peroxidation of membranes, decreased antioxidation capacity of cells due to decreased antioxidant enzyme activity, excessive release of nitric oxide, increased glutamic acid and downregulated levels of acetylcholine esterase activities [25].

During the present study, male mice exposed to a high dose of the nanocomposite had a significantly elevated catalase concentration compared to the control group (Table 4). Our results are in line with Zhao et al. [26], as they exposed mice to various lanthanide trichlorides and observed RRE accumulation in the kidney, leading to histopathological changes in the kidney, increased ROS production and lipid, DNA and protein peroxidation and disturbed catalase activities.

Oxidative stress also leads to lipid peroxidation of membranes and estimation of Malonaldehyde (MDA) is a standard indicator of membrane catastrophe [27]. MDA has a high affinity for thiol and amino groups of peptides, enzymes and nucleic acids and is extremely toxic to cells [28]. The present study showed that the MDA concentration in the liver and kidney was significantly higher in male mice exposed to the nanocomposite (Table 3). These results are in support of those from Huang et al. [22], who exposed mice to chlorides of various lanthanides, including Neodymium, and they observed a dose-dependent accumulation of RREs in hepatocytes and reported that catalase and SOD activities were decreased, while glutathione, glutathione peroxidase activity and malondialdehyde levels were elevated in hepatocytes. They concluded that oral administration of La, Ce and Nd leads to their accumulation in the nuclei and mitochondria of hepatocytes, resulting in oxidative stress.

## 5. Conclusions

In conclusion, we report that the two applied doses (10 and 20 mg/KgKg body weight) of the Neodymium Zirconate Zinc Sulfide nanocomposite significantly affect blood hemoglobin, serum cholesterol and triglyceride levels and biomarkers of oxidative stress in vital organs of albino mice of both sexes, but the effects were more pronounced in female mice and animals exposed to a lower dose of the nanocomposite.

## Figures and Tables

**Table 1:** Comparison of various studied parameters of blood count test between mice treated with Neodymium zirconate zinc sulfide nanocomposite, treated with low dose (10 mg/mL saline/kg body weight) and high dose (20 mg/mL saline/kg body weight) for 11 days with their respective untreated control groups. N = 8 for each treatment. Data are expressed as mean ± standard error of mean. *p*-value represents the results for two-sample *t*-test calculated for each studied parameter.

Experiment >	Low Dose (10 mg/mL solvent/kg body weight)				High Dose (20 mg/mL solvent/kg body weight)			
Parameters	Saline Treated Male	Neodymium Zirconate Zinc Sulfide Treated Male	Saline Treated Female	Neodymium Zirconate Zinc Sulfide Treated Female	Saline Treated Male	Neodymium Zirconate Zinc Sulfide Treated Male	Saline Treated Female	Neodymium Zirconate Zinc Sulfide Treated Female
Red blood cells (×10^6^ µL^−1^)	4.99 ± 0.51	6.23 ± 0.62	6.31 ± 0.80	6.12 ± 0.59	4.99 ± 0.51	6.51 ± 1.1	6.31 ± 0.80	5.97 ± 0.71
White blood cells (×10^3^ µL^−1^)	5.42 ± 1.6	4.25 ± 0.53	11.6 ± 3.9	13.4 ± 6.1	5.42 ± 1.6	10.75 ± 8	11.6 ± 3.9	8.85 ± 2.2
Hemoglobin (gdL^−1^)	10.97± 0.62	10.82 ± 0.31	10.61 ± 1.2	10.48 ± 0.83	10.97± 0.62	8.56 ± 0.9 *	10.61 ± 1.2	9.81 ± 1.1
Packed cell volume (%)	26.5 ± 4.7	31.5 ± 3.9	37.1 ± 5.5	34.7 ± 4.7	26.5 ± 4.7	33.7 ± 9.5	37.1 ± 5.5	34.5 ± 4.7
Mean corpuscular volume (fl)	52.0 ± 5.1	49.60 ± 2.8	57.60 ± 2.4	55.19 ± 3.4	52.0 ± 5.1	48.7 ± 5	57.60 ± 2.4	56.54 ± 2.2
Mean corpuscular hemoglobin (pg)	17.18 ± 0.23	18.07 ± 1.3	14.57 ± 2.1	17.43 ± 0.65	17.18 ± 0.23	19.06 ± 2.3	14.57 ± 2.1	16.56 ± 0.56
Mean corpuscular hemoglobin concentration (gdL^−1^)	34.50 ± 3.7	37.15 ± 3.6	24.8 ± 3.7	32.86 ± 3.1	34.50 ± 3.7	41.1 ± 7.0	24.8 ± 3.7	29.57 ± 1.5
Platelets (×10^3^ µL^−1^)	300 ± 72	388 ± 97	304 ± 41	335 ± 51	300 ± 72	360 ± 166	304 ± 41	347 ± 55
Lymphocytes (×10^3^ µL^−1^)	4.08 ± 1.1	3.583 ± 0.40	9.05 ± 2.5	8.5 ± 3.8	4.08 ± 1.1	7.6 ± 4.6	9.05 ± 2.5	6.78 ± 1.8
Monocytes (×10^3^ µL^−1^)	0.060 ± 0.024	0.050 ± 0.022	0.213 ± 0.072	0.263 ± 0.14	0.060 ± 0.024	0.280 ± 0.21	0.213 ± 0.072	0.163 ± 0.042
Granulocytes (×10^3^ µL^−1^)	1.24 ± 0.52	0.633 ± 0.15	2.41 ± 0.79	3.77 ± 2.4	1.24 ± 0.52	4.30 ± 3.2	2.41 ± 0.79	1.93 ± 0.40
Lymphocyte (%)	78 ± 5.1	84.60 ± 2.2	78.71 ± 2.1	76.42 ± 3.5	78 ± 5.1	73 ± 6.2	78.71 ± 2.1	75.24 ± 2.2
Monocytes (%)	1.140 ± 0.25	1.150 ± 0.18	17.63 ± 0.15	1.613 ± 0.18	1.140 ± 0.25	1.720 ± 0.29	17.63 ± 0.15	1.837 ± 0.19
Granulocytes (%)	20.9 ± 4.9	14.25 ± 2.1	19.52 ± 2.0	21.96 ± 3.3	20.9 ± 4.9	25.2 ± 6	19.52 ± 2.0	23.41 ± 2.3
Red cell distribution width (%)	24.90 ± 1.7	25.53 ± 2.6	12.25 ± 0.92	21.0 ± 0.74	24.90 ± 1.7	25.38 ± 3.5	12.25 ± 0.92	21.73 ± 1.2
Red cell distribution width -SD	43.98 ± 4.4	40.85 ± 3.0	47.1 ± 3.8	44.3 ± 3.9	43.98 ± 4.4	38.12 ± 3.8	47.1 ± 3.8	45.0 ± 1.8
Platelet distribution width (%)	13.98 ± 1.7	15.92 ± 1.9	13.6 ± 1.4	13.40 ± 1.1	13.98 ± 1.7	13.98 ± 0.75	13.6 ± 1.4	13.64 ± 1.4
Platelet crit	0.223 ± 0.047	0.275 ± 0.061	0.215 ± 0.03	0.237 ± 0.035	0.223 ± 0.047	0.249 ± 0.041	0.215 ± 0.03	0.249 ± 0.041
Mean platelet volume (fl)	7.28 ± 0.65	7.250 ± 0.39	7.087 ± 0.21	7.20 ± 0.42	7.28 ± 0.65	7.320 ± 0.44	7.087 ± 0.21	0.7287 ± 0.35

*p* > 0.05 = Non-significant; *p* < 0.05 = Least significant (*).

**Table 2:** Comparison of various studied parameters of serum biochemical analysis between mice with Neodymium zirconate zinc sulfide nanocomposite treated with low dose (10 mg/mL saline/kg body weight) and high dose (20 mg/mL saline /kg body weight) for 11 days with their respective untreated control groups. N = 8 for each treatment. Data are expressed as the mean ± standard error of the mean. *p*-values represent the results for two-sample *t*-test calculated for each studied parameter.

Experiment >	Low Dose (10 mg/mL solvent/kg body weight)				High Dose (20 mg/mL solvent/kg body weight)			
Parameters	Saline Treated Male	Neodymium Zirconate Zinc Sulfide Treated Male	Saline Treated Female	Neodymium Zirconate Zinc Sulfide Treated Female	Saline Treated Male	Neodymium Zirconate Zinc Sulfide Treated Male	Saline Treated Female	Neodymium Zirconate Zinc Sulfide Treated Female
Cholesterol mg/dL	156 ± 7.8	167.2 ± 7.5	154.2 ± 17	190.0 ± 4.7	156 ± 7.8	171.4 ± 5.2	154.2 ± 17	194.2 ± 1.7 *
Triglycerides mgdL^−1^	150.3 ± 24	162.0 ± 17	168.6 ± 7.5	219.2 ± 18 *	150.3 ± 24	151.9 ± 14	168.6 ± 7.5	222.1 ± 19 *
Creatinine mgdL^−1^	33.7 ± 28	16.1 ± 5.6	4.28 ± 2.4	7.10 ± 2.3	33.7 ± 28	14.2 ± 4.3	4.28 ± 2.4	10.8 ± 6.1
High density lipoprotein m	50.5 ± 5.4	49.6 ± 6.7	45.79 ± 3.0	45.60 ± 1.8	50.5 ± 5.4	61.6 ± 7.9	45.79 ± 3.0	50.3 ± 1.6
Low density lipoprotein mgdL^−1^	78.6± 11	88.7 ± 6.2	82.6 ± 13	102.1 ± 8.8	78.6± 11	78.1 ± 9.4	82.6 ± 13	91.35 ± 4.2

*p* > 0.05 = Non-significant; *p* < 0.05 = Least significant (*).

**Table 3:** Comparison of various studied antioxidant parameters between male/female albino mice supplemented orally for 11 days either with saline solution or 10 mg/mL solvent/KgKg body weight of Neodymium zirconate zinc sulfide nanocomposite. All values are expressed as mean ± slandered error of mean. *p*-value presents the results for the 2-sample *t*-test calculated for each parameter.

**Male**	**Parameter**	**Brain**			**Heart**			**Liver**			**Kidney**			**Lungs**		
		**Control**	**Treated**	***p* Value**	**Control**	**Treated**	***p* Value**	**Control**	**Treated**	***p* Value**	**Control**	**Treated**	***p* Value**	**Control**	**Treated**	***p* Value**
	Super oxide dismutase Unit/g	0.395± 0.22	3.32 ± 2.6	0.3	0.593 ± 0.21	0.742 ± 0.21	0.6	3.50 ± 1.5	9.01 ± 3.3	0.2	1.073 ± 0.32	2.75 ± 1.3	0.2	0.199 ± 0.080	2.66 ± 1.5	0.1
	Malonaldehyde Pico mol/g	160.3± 25	177.3 ± 19	0.6	464 ± 115	518 ± 85	0.7	169.2 ± 4.6	180.7 ± 2.8	0.05 *	375 ± 108	704 ± 81	0.03 *	366 ± 52	408 ± 71	0.6
	Catalase mg/dL	30.7 ± 0.21	30.5 ± 0.29	0.6	29.83 ± 1.3	30.47 ± 2.2	0.8	7.33 ± 1.5	13.0 ± 3.9	0.2	26.19 ± 2.0	29.15 ± 1.8	0.2	29.7 ± 3.8	34.18 ± 3.3	0.3
**Female**	**Parameter**	**Brain**			**Heart**			**Liver**			**Kidney**			**Lungs**		
		**Control**	**Treated**	***p* Value**	**Control**	**Treated**	***p* Value**	**Control**	**Treated**	***p* Value**	**Control**	**Treated**	***p* Value**	**Control**	**Treated**	***p* Value**
	Super oxide dismutase Unit/g	1.28 ± 0.74	2.49 ± 1.9	0.6	1.12 ± 47	0.865 ± 0.62	0.05 *	0.46 ± 0.18	4.58 ± 3	0.3	1.22 ± 0.40	0.806 ± 0.70	0.2	0.567 ± 0.096	0.768 ± 0.83	0.3
	Malonaldehyde Pico mol/g	138.1 ± 13	170.5 ± 5.4	0.07	103 ± 14	59.0 ± 22	0.1	174.6 ± 5.3	168 ± 2.7	0.3	121 ± 18	20 ± 81.2	0.1	60.6 ± 19	90.5 ± 25	0.3
	Catalase mg/dL	31.8 ± 0.4	31.6 ± 0.13	0.9	32.08 ± 1.3	27.6 ± 1.9	0.07	8.08 ± 0.9	10.04 ± 1.8	0.4	31.64 ± 1.9	31.86 ± 3.8	0.9	29.47 ± 2.7	28 ± 1.7	0.7

*p* > 0.05 = Non-significant; *p* < 0.05 = Least significant (*).

**Table 4:** Comparison of various studied antioxidant parameters between male/female albino mice supplemented orally for 11 days either with saline solution or 20 mg/mL saline/KgKg body weight of Neodymium zirconate zinc sulfide nanocomposite All values are expressed as mean ± slandered error of mean. *p*-values present the results for the 2 sample *t*-test calculated for each parameter.

**Male**	**Parameter**	**Brain**			**Heart**			**Liver**			**Kidney**			**Lungs**		
		**Control**	**Treated**	***p* Value**	**Control**	**Treated**	***p* Value**	**Control**	**Treated**	***p* Value**	**Control**	**Treated**	***p* Value**	**Control**	**Treated**	***p* Value**
	Super oxide dismutase Unit/g	0.395 ± 0.22	9.39 ± 3.0	0.05 *	0.593 ± 0.21	2.11 ± 1.5	0.3	3.50 ± 1.5	5.78 ± 2.2	0.4	1.073 ± 0.32	3.23 ± 1.2	0.1	0.199 ± 0.080	3.82 ± 2.1	0.1
	Malonaldehyde Pico mol/ g	160.3 ± 25	193.8 ± 17	0.3	464 ± 115	639 ± 69	0.2	169.2 ± 4.6	170.43 ± 3.3	0.8	3.75 ± 108	522 ± 86	0.3	366 ± 52	403 ± 43	0.5
	Catalase mg/dL	30.7 ± 0.21	31.0 ± 0.53	0.8	29.83 ± 1.3	29.3 ± 4.9	0.9	7.33 ± 1.5	5.24 ± 0.6	0.2	26.19 ± 2.0	36.6 ± 3.8	0.03 *	29.7 ± 3.8	31.9 ± 4.4	0.7
**Female**	**Parameter**	**Brain**			**Heart**			**Liver**			**Kidney**			**Lungs**		
		**Control**	**Treated**	***p* Value**	**Control**	**Treated**	***p* Value**	**Control**	**Treated**	***p* Value**	**Control**	**Treated**	***p* Value**	**Control**	**Treated**	***p* Value**
	Super oxide dismutase Unit/g	1.28 ± 0.74	1.83 ± 1.7	0.8	1.12 ± 47	02.41 ± 1.4	0.3	0.46 ± 0.18	0.61 ± 0.41	0.7	1.22 ± 0.40	248.6 ± 11	0.6	0.567 ± 0.096	21.4 ± 5.2	0.3
	Malonaldehyde Pico mol/ g	138.1 ± 13	145.4 ± 16	0.7	103 ± 14	65.7 ± 11	0.05 *	174.6 ± 5.3	174.3 ± 3.0	0.1	121 ± 18	114 ± 1.08	0.03 *	60.6 ± 19	58.2 ± 21	0.3
	Catalase mg/dL	31.8 ± 0.4	31.6 ± 0.24	0.8	32.08 ± 1.3	24.35 ± 2.5	0.1	8.08 ± 0.9	14.54 ± 3.1	0.07	31.64 ± 1.9	19.5 ± 3.7	0.01 **	29.47 ± 2.7	11.3 ± 4.0	0.7

*p* > 0.05 = Non significant; *p* < 0.05 = Least significant (*); *p* < 0.01 = Significant (**).

## Data Availability

All the data generated during this project are presented in the manuscript.
